# Male Infertility: Genetics, Mechanism, and Therapies

**DOI:** 10.1155/2016/7372362

**Published:** 2016-01-31

**Authors:** Charles Coutton, Rafael A. Fissore, Gianpiero D. Palermo, Katrien Stouffs, Aminata Touré

**Affiliations:** ^1^Institut Albert Bonniot, Université Grenoble Alpes, INSERM U823, CHU de Grenoble, UF de Génétique Chromosomique, 38000 Grenoble, France; ^2^Department of Veterinary and Animal Sciences, University of Massachusetts, Amherst, MA 01003, USA; ^3^Ronald O. Perelman and Claudia Cohen Center for Reproductive Medicine, Weill Cornell Medical College, New York, NY 10021, USA; ^4^Center for Medical Genetics, Universitair Ziekenhuis Brussel, 1090 Brussels, Belgium; ^5^INSERM U1016, Institut Cochin, CNRS UMR8104, Université Paris Descartes, Sorbonne Paris Cité, Faculté de Médecine, 75014 Paris, France

The World Health Organization declaimed that infertility is a major global public health issue of the last few decades. Infertility is commonly defined as the failure to conceive after 1 year of unprotected intercourse and is estimated to concern 72.4 million people worldwide with 40.5 million currently seeking medical care. The overall burden of subfertility/infertility is significant, is likely underestimated, and has not displayed any decrease over the last 20 years. Male factors are estimated to be involved, at least partially, in half of the cases. While the diagnosis, medical treatment, and psychosocial management of infertility have rapidly evolved over the past 4 decades, some difficulties still persist. Little is known about the physiopathology of altered sperm production, its genetic causes, or the genetic and epigenetic consequences for the gamete and the forthcoming conceptus. The information generated by conventional semen analysis has historically classified patients into categories lacking knowledge of causality and leaving conventional therapy as somewhat empirical. One of the reasons for this lack of fundamental understanding is the heterogeneity of causal factors as male infertility is a typical multifactorial disorder with a strong genetic basis and additional factors such as urogenital infections, immunological or endocrine diseases, attack from reactive oxygen species (ROS), or perturbations from endocrine disruptors. Since assisted reproduction technology (ART) is widely used to achieve conception with gametes produced by compromised spermatogenesis, there is a clear need to detail the molecular pathogenesis of male infertility to improve long-term risk assessment on a case-by-case basis. In this context, research on the male partner will shed a much-needed light on the physiopathology of male reproduction, will enhance patient management, and constitutes a prerequisite for the development of new therapeutic solutions.

